# Relação da Testosterona com a Obesidade e a Hipertensão Arterial

**DOI:** 10.36660/abc.20240138

**Published:** 2024-04-19

**Authors:** Rui Póvoa

**Affiliations:** 1 Universidade Federal de São Paulo São Paulo SP Brasil Universidade Federal de São Paulo, São Paulo, SP – Brasil

**Keywords:** Testosterona, Hipertensão, Obesidade

A testosterona é o principal hormônio masculino e é responsável pelo desenvolvimento dos órgãos sexuais, tendo importância na libido e na função sexual de forma geral. Apesar de controverso, acredita-se que com a idade ocorra diminuição gradativa deste hormônio, e alguns trabalhos apontam a obesidade como principal fator da redução hormonal.^1 , 2^

As diferenças hormonais no homem e na mulher podem explicar algumas peculiaridades no comportamento de determinadas doenças, principalmente as cardiovasculares (CV). A testosterona, atinge um pico aos 30 anos de idade, com declínio de 1-2% anualmente.^3^ Em idosos, o risco de apresentar deficiência de testosterona (DT) aumenta sensivelmente, podendo levar a diversos sintomas. Entretanto o efeito desta redução hormonal no risco CV ainda não está bem definido, pois em fases mais avançadas da vida surgem diversas comorbidades, principalmente a obesidade. Nesta mescla de fatores de risco fica um pouco difícil tipificar a importância da DT de forma objetiva. Alguns estudos como o de Laughlin et al.^4^ evidenciaram que a DT aumentava o risco CV. Outros como o " *The Longitudinal Cardiovascular Health Study* "^5^ e o de Collet et al.^6^ não encontraram nenhuma relação.

Apesar de não haver uma conclusão objetiva entre a DT e o risco CV, a terapia de reposição da testosterona (TRT) é amplamente utilizada, especialmente em idosos com baixos níveis deste hormônio e com alguma expressividade sintomática. Entretanto a TRT apresenta resultados discrepantes em relação aos eventos CV, podendo aumentar a mortalidade, apesar da significante melhora dos sintomas da deficiência androgênica.^7^

Esta discrepância, prejudicando uma conclusão dos estudos, ocorre devido a existência, nesta faixa etária mais avançada, de diversas comorbidades principalmente a obesidade, o diabetes mellitus, pré-existência de doenças cardiovasculares e principalmente a hipertensão arterial (HA). Em relação a HA alguns estudos associam a DT a aumentos dos níveis pressóricos, porém ainda não temos estudos robustos que confirmem esta afirmação.^8^

Este estudo transversal de Negreto et al.^9^ avaliou a prevalência da DT em uma população hipertensa tendo o grande mérito de ser uma avaliação inédita na população brasileira.^9^ A avaliação da DT em uma determinada doença é complexa em vista de diversos fatores de confusão. Em relação a HA, a obesidade é o fator de confusão mais presente visto que está relacionado tanto com a HA quanto com a DT. De todas as comorbidades analisadas neste estudo foi a obesidade a que teve a relação com a DT, com estatística significante. Encontraram nesta população hipertensa 24,5% de DT, entretanto não sabemos qual a real prevalência na população brasileira. Os estudos em outras populações apresentam números muito variáveis. Mulligan et al estudando o hipogonadismo em 2.165 indivíduos com idade acima de 45 anos (média de 60,5 anos), encontraram prevalência de 38,7%.^10^ Evidentemente que a composição das populações estudadas e a relação multidirecional entre a hipertensão, DT e aumento de peso corpóreo, impactam de forma expressiva em uma análise correta de qualquer uma destas variáveis. Entretanto, apesar dos possíveis vieses envolvendo estas doenças e comorbidades, a DT teve associação positiva como o índice de massa corpórea. Quanto mais obeso menor o nível de testosterona. Nesta população brasileira hipertensa, o nível de testosterona caiu um pouco mais de 8ng/dL para o aumento de 1Kg/m^2^ e reduziu 3,7 ng/dL para cada ano a mais de idade. Estes números são importantes na escassez de dados nacionais.

A participação dos hormônios no contexto cardiovascular é bastante ampla e ainda complexa, principalmente esta relação da testosterona com a HA, que é uma doença multifatorial, onde a obesidade participa de forma expressiva. A maioria dos fatores que elevam a pressão arterial estão relacionados com a obesidade e vice-versa, e neste cenário a DT se agrega a esta complexa dupla de fator de risco CV.

Ainda necessitamos mais estudos com a população brasileira para uma ideia da verdadeira prevalência da DT e para que possamos decifrar com precisão qual o papel do hormônio e da obesidade no mecanismo da HA.
