# Supplementary material for: Relação da Testosterona com a Obesidade e a Hipertensão Arterial
Source: Arq Bras Cardiol. 2024 Apr 19;121(3):e20240138. [Article in Portuguese] doi: 10.36660/abc.20240138 (PMC11081143; doi:10.36660/abc.20240138)
Supplement: Translation

# Translation: Relationship of Testosterone with Obesity and Arterial Hypertension

**DOI:** 10.36660/abc.20240138i

**Authors:** Rui Póvoa

**Affiliations:** 1 São Paulo SP Brazil Universidade Federal de São Paulo, São Paulo, SP – Brazil

**Keywords:** Testosterone, Hypertension, Obesity

Testosterone is the main male hormone and is responsible for the development of sexual organs, having importance in libido and sexual function in general. Although controversial, it is believed that this hormone gradually decreases with age, and some studies point to obesity as the main factor in hormonal reduction.^1 , 2^

Hormonal differences in men and women may explain some peculiarities in the behavior of certain diseases, especially cardiovascular (CV) diseases. Testosterone reaches a peak at 30 years of age, with a decline of 1-2% annually.^3^In the elderly, the risk of developing testosterone deficiency (TD) increases significantly, which can lead to several symptoms. However, the effect of this hormonal reduction on CV risk is not yet well defined, as various comorbidities arise at later stages of life, especially obesity. In this mix of risk factors, it is somewhat difficult to typify the importance of DT objectively. Some studies, such as that by Laughlin et al.,^4^showed that DT increased CV risk. Others, such as "The Longitudinal Cardiovascular Health Study"^5^and that of Collet et al.^6^found no relationship.

Although there is no objective conclusion between TD and CV risk, testosterone replacement therapy (TRT) is widely used, especially in elderly people with low levels of this hormone and with some expressive symptoms. However, TRT presents discrepant results concerning CV events and may increase mortality, despite the significant improvement in androgen deficiency symptoms.^7^

This discrepancy, hindering the conclusion of the studies, occurs due to the existence, in this older age group, of several comorbidities, mainly obesity, diabetes mellitus, pre-existence of cardiovascular diseases, and mainly arterial hypertension (AH). About AH, some studies associate DT with increased blood pressure levels, but we still do not have robust studies that confirm this statement.^8^

This cross-sectional study by Negreto et al.^9^evaluated the prevalence of TD in a hypertensive population, having the great merit of being an unprecedented assessment of the Brazilian population.^9^The assessment of TD in a given disease is complex because of several confounding factors. Concerning AH, obesity is the most common confounding factor as it is related to both AH and TD. Of all the comorbidities analyzed in this study, obesity was the one that had a statistically significant relationship with TD. They found 24.5% of DT in this hypertensive population, however, we do not know the real prevalence in the Brazilian population. Studies in other populations present very variable numbers. Mulligan et al., studying hypogonadism in 2,165 individuals aged over 45 years (average of 60.5 years), found a prevalence of 38.7%.^10^Evidently, the composition of the populations studied and the multidirectional relationship between hypertension, TD, and increase in body weight, have a significant impact on a correct analysis of any of these variables. However, despite the possible biases involving these diseases and comorbidities, DT had a positive association with body mass index. The more obese you are, the lower your testosterone level. In this hypertensive Brazilian population, the testosterone level fell a little more than 8ng/dL for an increase of 1kg/m^2^and reduced by 3.7 ng/dL for each additional year of age. These numbers are important given the scarcity of national data.

The role of hormones in the cardiovascular context is quite broad and still complex, especially this relationship between testosterone and AH, which is a multifactorial disease, in which obesity plays a significant role. Most of the factors that increase blood pressure are related to obesity and vice versa, and in this scenario, TD is added to this complex duo of CV risk factors.

We still need more studies with the Brazilian population to get an idea of the true prevalence of TD so that we can decipher precisely the role of the hormone and obesity in the mechanism of AH.
